# Applying the Theoretical Domains Framework to Develop an Intervention to ‘Re-implement’ Parent–Child Interaction Therapy (PCIT)

**DOI:** 10.1007/s10488-023-01298-3

**Published:** 2023-09-10

**Authors:** Melanie J. Woodfield, Sharon T. Phillips, Tania Cargo, Sally N. Merry, Cheryl B. McNeil, Sarah E. Hetrick

**Affiliations:** 1 Te Ara Hāro-Centre for Infant, Child and Adolescent Mental Health, Department of Psychological Medicine, University of Auckland, Private Bag 92019, Auckland Mail Centre, Auckland, 1142 New Zealand; 2 Te Whatu Ora (Health New Zealand), Te Toka Tumai Auckland, Auckland, New Zealand; 3 Department of Psychology, University of Auckland, Auckland, New Zealand; 4 Centre for Youth Mental Health, University of Melbourne, Melbourne, Australia; 5 Department of Psychology, West Virginia University, Morgantown, USA; 6 Department of Psychiatry, University of Florida, Gainesville, USA

**Keywords:** Sustainment, Sustainability, Parent–child interaction therapy, PCIT, Theoretical domains framework, COM-B

## Abstract

Parent–Child Interaction Therapy (PCIT) is an empirically supported treatment for childhood conduct problems, with increasing numbers of clinicians being trained in Aotearoa/New Zealand. However, ensuring sustained delivery of effective treatments by trained clinicians in routine care environments is notoriously challenging. The aims of this qualitative study were to (1) systematically examine and prioritise PCIT implementation barriers and facilitators, and (2) develop a well specified and theory-driven ‘re-implementation’ intervention to support already-trained clinicians to resume or increase their implementation of PCIT. To triangulate and refine existing understanding of PCIT implementation determinants from an earlier cross-sectional survey, we integrated previously unanalysed qualitative survey data (54 respondents; response rate 60%) with qualitative data from six new focus groups with 15 PCIT-trained clinicians and managers in Aotearoa/New Zealand. We deductively coded data, using a directed content analysis process and the Theoretical Domains Framework, resulting in the identification of salient theoretical domains and belief statements within these. We then used the Theory and Techniques Tool to identify behaviour change techniques, possible intervention components, and their hypothesised mechanisms of action. Eight of the 14 theoretical domains were identified as influential on PCIT-trained clinician implementation behaviour (Knowledge; Social/Professional Role and Identity; Beliefs about Capabilities; Beliefs about Consequences; Memory, Attention and Decision Processes; Environmental Context and Resources; Social Influences; Emotion). Two of these appeared to be particularly salient: (1) ‘Environmental Context and Resources’, specifically lacking suitable PCIT equipment, with (lack of) access to a well-equipped clinic room appearing to influence implementation behaviour in several ways. (2) ‘Social/Professional Role and Identity’, with beliefs relating to a perception that colleagues view time-out as harmful to children, concerns that internationally-developed PCIT is not suitable for non-Māori clinicians to deliver to Indigenous Māori families, and clinicians feeling obligated yet isolated in their advocacy for PCIT delivery. In conclusion, where initial implementation has stalled or languished, re-implementation may be possible, and makes good sense, both fiscally and practically. This study suggests that re-implementation of PCIT in Aotearoa/New Zealand may be facilitated by intervention components such as ensuring access to a colleague or co-worker who is supportive of PCIT delivery, access to suitable equipment (particularly a time-out room), and targeted additional training for clinicians relating to the safety of time-out for children. The feasibility and acceptability of these intervention components will be tested in a future clinical trial.

**Supplementary Information:**

The online version contains supplementary material available at 10.1007/s10488-023-01298-3.

## Background

Internationally, disruptive behaviour disorders are one of the most frequent mental disorders formally diagnosed in children under 7 years (Vasileva et al., 2020). They represent around half of all childhood psychopathology and are one of the more common reasons for young children to be referred to mental health services (Coghill, 2013; Scott & Gardner, 2015). Parent–Child Interaction Therapy (PCIT; Eyberg, 1988) is an empirically supported treatment for disruptive behaviour in 2–7 year old children (Lieneman et al., 2017; Thomas et al., 2017; Ward et al., 2016; Zimmer-Gembeck et al., 2018). It is distinctive from many parent training interventions in that a clinician provides live coaching to a caregiver as they interact with their child, usually by way of an earpiece, through a one-way mirror. PCIT includes two phases: relationship enhancement (Child Directed Interaction; CDI) and behaviour management (Parent Directed Interaction; PDI). The CDI phase supports the parent to engage in daily child-led play, along with delivering specific skills designed to enhance the parent–child relationship (Eyberg & Funderburk, 2011; McNeil & Hembree-Kigin, 2011). The PDI phase teaches parents to use effective commands, and to follow through with a consequence such as time-out if required (Eyberg & Funderburk, 2011). In the clinic setting, a back-up room or separate space is used for time-out as a brief consequence for the child not remaining on a time-out chair as instructed (Eyberg & Funderburk, 2011).

PCIT has been shown to be effective in reducing child conduct problems, improving children’s compliance, decreasing parent stress, and improving parent emotion regulation and reflective functioning (Lieneman et al., 2017; Thomas et al., 2017; Ward et al., 2016; Zimmer-Gembeck et al., 2018). To become accredited in delivering PCIT, a Masters-level clinician completes a 40-h training, followed by 12 months of fortnightly hour-long group PCIT consultation, along with two completed PCIT cases (PCIT International Inc, 2018).

PCIT was developed in the USA, and the first cohort of clinicians were trained in Aotearoa/New Zealand in 2010. At the time of this study, approximately 135 clinicians had completed the initial 40-h training. PCIT-trained clinicians are located within a number of different service delivery contexts, predominantly child welfare services and government-funded child mental health services (Woodfield et al., 2020). Agencies are required to endorse the clinician attending initial PCIT training and undertake to support their use of PCIT post-training, however there is no formal expectation that the agency will provide equipment (e.g., a one-way mirror and parent ear-piece), nor is there a formal requirement for the agency to guarantee suitable PCIT clients will be available to the clinician post-training. A recent survey of PCIT-trained clinicians in Aotearoa/New Zealand found that 51.4% had changed agency or service since their initial PCIT training, with 10.7% of clinicians having changed agency twice or more (Woodfield et al., 2020), and as such, even where resources are available to the clinician at the time of their initial PCIT training, this may change over time.

At the time of this study, as few as 24% of PCIT-trained clinicians in Aotearoa/New Zealand reported using PCIT in their work (Woodfield et al., 2020). The majority of clinicians who were delivering PCIT reported seeing a modest number of clients per week (typically one or two clients), and these were commonly co-worked with another clinician, so potentially ‘counted’ twice (Woodfield et al., 2021). Despite this, even those clinicians who were not delivering PCIT typically described it as effective and broadly acceptable as a treatment (Woodfield et al., 2021).

### Aotearoa: Cultural Context

Māori are the Indigenous people of Aotearoa/New Zealand, and comprise 17.1% of the overall population, with a younger median age (Statistics New Zealand, 2021). A treaty (Treaty of Waitangi/Te Tiriti o Waitangi) was signed in 1840 between Māori and colonial settlers. The principles of the treaty, as articulated by the Courts and the Waitangi Tribunal, set out responsibilities and obligations, including for health providers to facilitate Māori self-determination, equity, protection and partnership (Manatū Hauora/Ministry of Health, 2020). These considerations are imperative given that the process of colonisation of Aotearoa is described as having “dispossessed Māori of 95 percent of their lands and resources, usurped Māori power and authority and left them in a state of poverty, deprivation and marginalisation while procuring considerable wealth, prosperity and privilege for British settlers” (Mutu, 2019). The process of colonisation, alienation from the land, and experiences of systemic racism continues to influence Māori wellbeing both directly and indirectly, for example, rates of childhood conduct problems are over twice as high in Māori children than non- Māori (Advisory Group on Conduct Problems, 2011; Cargo, 2020). Findings from a recent mixed methods study carried out by the Māori national PCIT trainer for Aotearoa/New Zealand, Dr Tania Cargo, suggested that standard, manualised PCIT may be acceptable to, and effective for, Māori families when a ‘by Māori, for Māori’ approach is utilised (Cargo, 2020), i.e., that PCIT is delivered to Māori families by Māori clinicians where possible.

### Sustainability in Community Settings

The effectiveness of a treatment does not guarantee its implementation in routine care environments (Bauer & Kirchner, 2020). In fact, the context within which treatments are delivered exerts a strong influence both on initial implementation (Kaplan & Walsh, 2022) and sustained delivery after the withdrawal of early implementation training and supports (Shoesmith et al., 2021). Findings from a recent systematic review suggested that research attention has predominantly been focused on PCIT implementation immediately post-training, and/or differential initial training methods to enhance downstream implementation (Woodfield et al., 2022). Efforts to increase initial adoption of PCIT by clinicians post-training have included remotely delivered co-therapy (Funderburk et al., 2015), learning collaboratives (Herschell et al., 2015), and system-level or state-wide initiatives (Herschell et al., 2018).

The sustainability of treatments in real world contexts is a relatively new area of focus for the field of implementation science (Herlitz et al., 2020), as is the concept of intervening to promote ‘re-implementation’ where implementation has stalled or ceased (Woodfield et al., 2021). To set the scene for sustained delivery, ideally agencies would ensure availability of necessary equipment and appropriate referral streams prior to a clinician undertaking PCIT training. However, this may not occur for several reasons, including funding only being available for initial training, or clinicians relocating to another agency setting (Timmer et al., 2016). We suggest that re-implementation interventions may be initiated months or years after a clinician is trained in the delivery of a clinical intervention to capitalise on the initial training investment that has already occurred. Re-implementation may be worthwhile attempting, as if delivery of evidence-based treatments is not sustained, it may be wasteful of the initial implementation investment, and potential clinical and public health benefits may not be realised (Nathan et al., 2022).

The influence of context on clinician implementation behaviour occurs both across and within a number of dimensions—including the outer setting (wider community, system, or country), the inner setting (hospital, clinic, or school), the innovation itself (treatment, programme or service), the implementation process (activities or strategies undertaken to implement the treatment) and the individuals charged with implementing or delivering the treatment (both their roles, and their characteristics) (Damschroder et al., 2022). While there are multiple layers to consider, understanding influences on the behaviour of these “deliverers” (Damschroder et al., 2022) is central to implementation success, as “implementation almost always requires someone or groups of individuals to do something differently” (Lorencatto, 2022). Interventions to change deliverer behaviour will be more effective when the behaviour itself is comprehensively understood, and based on accurate assumptions about what needs to change (Michie et al., 2014).

### Theoretical Domains Framework

Understanding deliverer behaviour requires undertaking a systematic process of identifying and prioritising the barriers (and facilitators) they experience (Weiner et al., 2022). There have also been calls to extend and deepen this understanding by better specifying both causal mechanisms of these determinants (e.g., *how* a particular barrier influences clinician implementation behaviour), and the hypothesised mechanisms of action of any implementation strategies to address these barriers (Lewis et al., 2018, 2020; Sales et al., 2021).

The Theoretical Domains Framework (TDF) is one of the more prominent frameworks that provides a system to organise barriers and facilitators, also known as determinants of implementation (Schleider & Beidas, 2022). It is associated with the Behaviour Change Wheel, a practical tool which supports moving beyond simply identifying barriers and facilitators, to constructing bespoke interventions in a structured and empirically supported manner (Michie et al., 2014). A strength of the TDF is the focus on change mechanisms that can readily translate into targets for implementation strategies (Schleider & Beidas, 2022). The TDF is comprised of 14 domains that comprehensively encapsulate possible influences on behaviour: Knowledge, Skills, Social/Professional Role and Identity, Beliefs about Capabilities, Optimism, Beliefs about Consequences, Reinforcement, Intentions, Goals, Memory, Attention and Decision Processes, Environmental Context and Resources, Social Influences, Emotions, and Behavioural Regulation (Cane et al., 2012). It has been used to understand the influences on deliverer behaviour, and develop targeted interventions, in numerous studies (for example, Barker et al., 2016; Brierley et al., 2021; Castro et al., 2021; Courtenay et al., 2019; Gould et al., 2017; Kierkegaard et al., 2021; Patey et al., 2012). The TDF is linked to a well-established model of behaviour, the COM-B, which suggests that Behaviour occurs as an interaction between three necessary conditions: Capability, Opportunity and Motivation (Michie et al., 2011). Clinicians only perform a behaviour when they have both the (perceived or actual) Capability and Opportunity, and when they are more Motivated to perform that behaviour than any other behaviour (West & Michie, 2020). In order for Motivation (which both directs and energises a behaviour) to generate a behaviour, both ‘gates’ of Opportunity and Capability need to be open (West & Michie, 2020). Capability may be physical or psychological (i.e., may relate to physical functioning and/or mental functioning); Opportunity may be social or physical (i.e., may relate to culture or social norms, and/or financial or material resources); and Motivation may be reflective or automatic (i.e., may involve conscious thought processes, or desires and habits) (West & Michie, 2020).

Michie et al. (2014) recommend a systematic intervention design or ‘behavioural diagnosis’ process to understand the implementation behaviour, and shape a behaviour change intervention. This includes initially defining the problem in behavioural terms, or “*who* needs to do *what* differently *to/for whom* and *when*” (Gillies et al., 2021). The AACAT framework may be used to specify the behaviour of individuals within implementation research, according to the Actor, Action, Target, Context, and Time domains (Gillies et al., 2021; Presseau et al., 2019). In this study, the specific implementation behaviour of interest is Aotearoa/New Zealand-based clinicians who have previously completed a 40-h PCIT training (*Actor*) delivering PCIT with fidelity to Eyberg and Funderburk’s (2011) treatment protocol (*Action*) to children aged 2–7 years with disruptive behaviour and their families (*Target*) in the service/agency/practice where children and families are routinely seen (*Context*), every time a suitable client presents (*Time*). Table 1 orients the present study in the context of our earlier work, according to the behavioural diagnosis process.

**Table 1:**
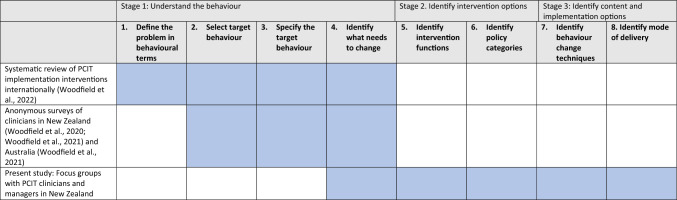
Focus of the current study, in the context of the wider research programme

Atkins et al. (2017) suggested that the validity of a behavioural diagnosis is enhanced through triangulation of data. This study will triangulate and refine our existing understanding of PCIT implementation determinants by applying the TDF to previously unanalysed qualitative survey data and new focus group data. The aims are to (1) systematically examine and prioritise implementation barriers and facilitators, and (2) develop a well-specified and theory-driven re-implementation intervention, along with hypothesised mechanisms of action, to support already-trained clinicians to resume implementing PCIT.

## Methods

### Participants

Data for the present study were drawn from two sources, namely (1) previously unanalysed qualitative data from an online survey and (2) new qualitative data from focus groups.

1. Participants in the *online cross-sectional survey* included clinicians based in Aotearoa/New Zealand or Australia who had completed PCIT training, i.e., a PCIT International 40-h training or its equivalent (refer Woodfield et al., 2021). Participating clinicians were not required to have achieved accreditation in PCIT. They were recruited via an email invitation circulated to PCIT-trained clinicians in NZ and Australia in mid-2021.
2. Participants in the *focus groups* were either (a) clinicians who had completed an initial PCIT training, as above, and/or (b) managers or funders of services where PCIT was delivered. Invitations to participate were circulated via email to an existing list of PCIT-trained clinicians in Aotearoa/New Zealand compiled for an earlier study (Woodfield et al., 2020). A small number of managers of services where PCIT was delivered were purposively sampled and approached directly via email. Participants were provided with a detailed information sheet, outlining the reasons for the study.

### Sample Size

Regarding the *online survey free-text responses*, 54 responses (a response rate of 60%) were received from PCIT-trained clinicians in Aotearoa/New Zealand (see Woodfield et al., 2021 for details).

Regarding the *focus groups*, published guidance for conducting TDF-based implementation research suggests that both individual interviews and focus groups may be appropriate, and that a key consideration is the stage of investigation (Atkins et al., 2017). In this study, we elected to use focus groups, as we sought to develop and deepen our earlier survey-based insights and understand any disagreements in participant perspectives. In terms of the number of groups, a minimum of three groups is recommended if the focus is on a specific implementation setting (Atkins et al., 2017), and more if the intervention is being implemented in a variety of settings. In our study, pragmatic considerations informed the sample size for the focus groups. The population of PCIT-trained clinicians in Aotearoa/New Zealand was approximately 135 at the time, and we estimate that the invitation to participate reached approximately 90 clinicians. A COVID-19 related lockdown was in place at the time of the study. An initial invitation, followed by one reminder was acceptable to the ethics committee, and all clinicians who responded were included in a focus group.

### Materials

Regarding the online survey, the rationale for included items is described elsewhere (see Woodfield et al., 2021). In brief, the survey contained Likert scales, and other quantitative data (see Woodfield et al., 2021). Qualitative data from a series of open-ended or free-text items (available from the first author) had not previously been analysed. The facility to provide free-text responses was only available in relation to selected items, including “If you have persisted with using PCIT, why have you done so (i.e., what has sustained you in your PCIT work)?” and “If you no longer use PCIT, what would have made it easier to continue to use PCIT in your work?” and “Finally, is there anything else that you would like to share about your experience of using PCIT in your work?”. Responses to these items from Aotearoa/New Zealand-based participants, were integrated with new data drawn from focus groups.

Regarding the focus groups, interview schedules (a version for clinicians, and a slightly modified version for managers/funders) were developed based on the quantitative findings from the earlier online survey, with the intention of more comprehensively investigating and triangulating previously identified barriers and facilitators to PCIT implementation from the analysis of the survey data. In this quantitative survey data, the most highly ranked facilitators of adoption of PCIT by clinicians were access to a suitably equipped clinic room, and the ability to co-work with another clinician (Woodfield et al., 2021). The most highly ranked barriers were lacking suitable equipment, families struggling to attend clinic-based sessions, and families’ discomfort with the Parent-Directed Interaction phase (which includes time-out) (Woodfield et al., 2021).

In the focus groups, the interview schedules were used as an initial prompt for the facilitator (the first author; MW, an experienced Doctoral-level female clinical psychologist, who is PCIT-trained), who prioritised probing participants’ comments—stated plainly, to understand *why* and *how* these factors acted as barriers. Probing questions included “How, or in what way, might that make a difference?”, “How confident would you be that this would make a difference to whether clinicians start using PCIT again?”, and finally an open-ended prompt of “Is there anything we haven’t covered that you think is important?”.

### Procedure

Approval for the study was obtained from the Auckland Health Research Ethics Committee (Ref: AH22277). Approval for sharing anonymised data with authors SP and CMcN was also obtained from AHREC and occurred via a data sharing agreement between the Universities of Auckland and West Virginia. Focus groups were conducted during October and November 2021, online via Zoom, owing to COVID-19 related lockdowns in Aotearoa/New Zealand at the time. At the beginning of the Zoom meeting, a brief Zoom poll collected basic demographic characteristics from participants. Each group was recorded. Only participants and the facilitator were present in the meeting. The Zoom-generated audio transcript was used as a basis for verbatim transcription, and was reviewed, cleaned and edited by the first author (MW). Transcripts were anonymised (pseudonyms assigned; any identifying material removed) prior to sharing with other study team members.

### Data Analysis

#### Clarifying and Prioritising Barriers and Enablers

Using a directed content analysis process (Atkins et al., 2017), MW initially deductively coded the qualitative data into all of the 14 Theoretical Domains using NVivo software (QSR International Pty Ltd. 2020). Cane et al. (2012)’s definitions of the Theoretical Domains were used to guide the coding, along with the domain-related questions outlined in Michie et al. (2014) and guidance provided by Atkins et al. (2017). During coding, MW kept field notes and created a codebook to describe how data from this study were attributed to the Domains. “Belief statements” within each domain, i.e., “a collection of responses with a similar underlying belief that suggest a problem and/or influence of the beliefs on the target implementation problem” (Atkins et al., 2017) were initially generated by MW using an inductive thematic analysis approach (Clarke & Braun, 2013) and subsequently shared with a second author (SP), who reviewed all transcripts and coding, for consistency, accuracy and validity. MW and SP then met to discuss coding disagreements and reach consensus relating to which domains initially appeared to exert the most salient influence on clinician behaviour. Inter-coder reliability was not calculated, as coding in this phase was intended to produce the ‘building blocks’ for the synthesis, conceptualisation, and integration with theory that subsequently occurred with the wider research team (O’Connor & Joffe, 2020). Also, other studies following a similar methodology (e.g., Patey et al., 2012) have presented frequency counts of belief statements at this point, however this is less relevant for data drawn from focus groups, as social influence may elicit more agreement than in individual interviews (Atkins et al., 2017).

The relevant Theoretical Domains and their associated belief statements were then shared and discussed with the wider research team, in order to determine possible behaviour change intervention targets (Atkins et al., 2017). Atkins et al. (2017) suggest that relevant or salient domains, i.e., domains which should be targeted in an intervention, may be identified by the research team in several ways. As outlined earlier, a simple frequency count of data coded into each domain is not appropriate for focus group studies (Atkins et al., 2017). In published studies using the TDF, the importance, salience, or relevance of particular domains as intervention targets has involved concurrent consideration of several factors in this refinement of possible intervention foci, including the presence of any conflicting beliefs, and the perceived strength and influence of the beliefs on clinician behaviour (Atkins et al., 2017). In our study, this discussion drew on the research team’s knowledge of PCIT, Māori cultural considerations, previous research, and observations and experiences in clinical settings. This process is in keeping with the methodology recommended by TDF guidance such as that provided by Atkins et al. (2017).

#### Identifying Behaviour Change Techniques

The online Theory and Techniques Tool (see https://theoryandtechniquetool.humanbehaviourchange.org/tool) allows for links between 74 behaviour change techniques and 26 mechanisms of action to be understood and specified (Carey et al., 2019; Connell et al., 2019; Johnston et al., 2021). This tool was used to identify evidence-based behaviour change techniques associated with each of the Theoretical Domains (i.e., mechanisms of action) identified as relevant and salient in this context.

## Results

Aotearoa/New Zealand participants comprised 60% of the total number of respondents to the survey, and further details and demographic characteristics are reported in Table 1 of Woodfield et al. (2021). In brief, respondents predominantly identified as New Zealand European (70%), and 15% (8 of 54 respondents) identified as Māori. Participating clinicians were predominantly female (93%), and had a professional background as a psychologist (70%).

Regarding the focus groups, 15 participants attended six focus groups (group sizes ranged from 1 to 3 participants) in late 2021, as outlined in Table 2. One focus group contained only a single participant due to the unexpected unavailability of other group members. Participating clinicians (n = 12) and managers/funders (n = 3) were predominantly female (n = 12), and located within Infant, Child and Adolescent Mental Health Services (n = 9). Focus groups ranged from 35 to 60 min in length, with most being 50–60 min long.

**Table 2:** Demographic characteristics of focus group participants

Group	Participant ethnicity	Gender	Profession	Time since PCIT training	Current service/agency
1	Māori	Female	Social Worker; Other	4–6 years ago	Infant, Child and Adolescent Mental Health Service (ICAMHS); Private practice
	New Zealand European	Female	Clinical Psychologist, Psychologist or Trainee Psychologist	4–6 years ago	Infant, Child and Adolescent Mental Health Service (ICAMHS)
	New Zealand European	Female	Clinical Psychologist, Psychologist or Trainee Psychologist	4–6 years ago	University clinic
2	Other	Male	Manager (Occupational Therapist)	N/A	Infant, Child and Adolescent Mental Health Services (ICAMHS)
	New Zealand European	Female	Manager (Nurse)	N/A	Not currently working in a clinical role
	New Zealand European	Female	Funder (Clinical Psychologist, Psychologist or Trainee Psychologist)	N/A	Not currently working in a clinical role
3	New Zealand European	Male	Clinical Psychologist, Psychologist or Trainee Psychologist	2–4 years ago	Infant, Child and Adolescent Mental Health Service (ICAMHS)
	New Zealand European	Female	Nurse	More than 6 years ago	Infant, Child and Adolescent Mental Health Service (ICAMHS)
4	New Zealand European	Female	Social Worker	0–2 years ago	Infant, Child and Adolescent Mental Health Service (ICAMHS)
	New Zealand European	Female	Other	4–6 years ago	Child protective services
	New Zealand European	Female	Clinical Psychologist, Psychologist or Trainee Psychologist	4–6 years ago	Infant, Child and Adolescent Mental Health Service (ICAMHS)
5	New Zealand European	Male	Clinical Psychologist, Psychologist or Trainee Psychologist	More than 6 years ago	Child protective services
	New Zealand European	Female	Counsellor	More than 6 years ago	Infant, Child and Adolescent Mental Health Service (ICAMHS)
	New Zealand European; Other	Female	Social Worker	0–2 years ago	Child protective services
6	New Zealand European	Female	Clinical Psychologist, Psychologist or Trainee Psychologist	0–2 years ago	Infant, Child and Adolescent Mental Health Service (ICAMHS)

### Clarifying and Prioritizing Barriers and Enablers

Of the 14 possible constructs from the Theoretical Domains Framework, eight were identified as being relevant influences on clinician behaviour appropriate to be targeted with an intervention. These included Knowledge; Social/professional role and identity; Beliefs about capabilities; Beliefs about consequences; Memory, attention and decision processes; Environmental context and resources; Social influences; and Emotion. These domains, along with the belief statements that were generated within each domain, and quotes to illustrate these, are presented in Table 3.

**Table 3:** Relevant TDF domains, associated belief statements, and illustrative quotes

Domain	Belief statements	Illustrative quotes
Knowledge	• Training is hard to access—eligibility criteria, funding, and availability • The manualised protocol is restrictive and inflexible • Yet the structure of the manual can be supportive, especially when learning PCIT • I know that PCIT has a strong evidence-base	“*Sometimes it seems perfect and other times you want to do things differently, but then… you feel like you're not being adherent*” (Group 1, clinician) “*…it is manualised and it is very structured and it’s very kind of you know, if we stick to that, then you can't go wrong? I mean there are times… where you do have to make … modifications and especially if it's not working, and you don't see a change.*” (Group 1) “*What needs to be in place, yeah… access to the training. Because, as you’re aware, CAMHS workforces are incredibly fluid just now*” (Group 2, manager) “*say I’d never heard of PCIT and somebody came and said *“*I’ve done a bit of Googling about this, it seems like a good idea*”*. You know the first thing I’d be asking *“*what's, what's the evidence?*”” (Group 2, manager) “*PCIT has, um you can lean on the manual quite significantly? And especially at those initial stages?*” (Group 3, clinician) “*the risk of kind of getting it wrong somehow, or not quite following… [the] protocol, right? There's a manual. But it never goes… [to plan]. So there's always, I guess that tension, for me, sometimes around how am I going to manage whatever comes up. Because you've got to be able to go with the moment, you've got to be able to manage that, and be confident about the protocol so that you can have that flexibility and respond in the moment*” (Group 4, clinician) “*…not just doing what you think and using your clinical judgment, but making sure you're following this jolly prescriptive protocol, which is very… kind of rigid, if you like. So I think there's those two pressures. Get it right, be effective, be responsive in the moment to this little person and this distressed parent. And not only do that, make sure you follow this recipe which you've got to do right.*” (Group 4, clinician) “… *while we’re learning we’re going to really stick to the words, be really thorough, like do the full Monty? You know, I’m a psychologist we all have a bit of that sort of, you know… following the rules. And on this occasion, I mean I can get a bit fluffy at times. But the research is based on this is what, how we have to do it. So we both decided we needed to keep each other… true to the model.*” (Group 6, clinician) “*I also find the branding of therapies that require total compliance a little frustrating. Adherence to a manualised intervention ultimately runs counter to formulation based practice*” (survey respondent) “*There's very little demonstrated stuff for under 8 s (pause) that, that has such compelling evidence as PCIT. So I mean, I have no problem supporting it in the, in the service that that I manage.*” (Group 2, manager)
Social/professional role and identity**	• Regarding PDI, I’m concerned about the views of, and impact on my colleagues who are not trained in PCIT. My colleagues believe that time-out is harmful for children • I need to advocate for PCIT within the service, often alone. To convince managers and colleagues of both its safety and usefulness • A supportive manager, and a supportive team, is essential • I worry that it’s not a good fit for Māori families. If I am not Māori, I should not work with Māori families	“*at the beginning it's really great to have somebody else there with you when you're new and it's all pretty scary, especially the first PDI I wouldn't want to do that on my own (laughs). But, yeah I think the co-working for me is also about having that support, even if they're not in the room with you? So having… somebody within your agency or workplace that is there with you. Helping you advocate for PCIT… once you've got your head around actually the mechanics of PCIT, it doesn't necessarily have to be a co-worker in the room but I think you need that professional support within your workplace.*” (Group 1, clinician) “*if you’re having to be the lone… you have 125% believe that this is the only way, right? (laughs) Um yeah otherwise it's like *“*okay well it's probably easier just to… do Incredible Years with this family*”* because you're having to put so much into advocating for this.*” (Group 1, clinician) “*I had some very difficult conversations with managers who you know, *“*Would you actually use it? How many people do you think you’d have on your books?*”* and all of these kind of things. So that… (pause) Being on the other side of that now, there is part of me that thinks, I’m not sure if I would be willing to go through that again. Although I really love the intervention.*” (Group 3, clinician) “*I mean there's a lot of… opposition to PCIT. There's a lot of *“*is this traumatic for the child*”* and just a visceral kind of like a turnoff (laughs) I think [colleagues] just don't want to be around… you know, imposing distress on a child?*” (Group 3, clinician) “*[PCIT] was new to New Zealand, I think. Or fairly new. And I remember my colleagues, that visceral response was just like *“*no way, that's just terrible, we can't, you know I just can't…*”* yeah it was quite strong.*” (Group 3, clinician) “*It absolutely would probably be the thing that I hear the most—and for myself—puts [colleagues] off. *“*I don't want to do PCIT because I hate that time-out thing*”* is what I hear. And, like other clinicians and staff freaking out with the noise of the kids screaming and banging. I mean that's a logistical issue, which is a barrier… it’s just like *“*Urgh! Do I really want to go through that (laughs), even though I know in my heart this approach works…*”” (Group 4, clinician) “*If you don't have a supportive manager it's just not going to happen. I think that's probably, and the manager might have some really strong opinions around something like PCIT.*” (Group 3, clinician) “*Manager support. If you don't have that you're screwed.*” (Group 4, clinician) “*And I wouldn't want to be fighting management and fighting all the way. I’d want, I really want to work somewhere where it was seen as a really valid, important thera… intervention. And then I’d be in like Flynn!*” (Group 6, clinician) “*my culture has on occasion has been a barrier to me providing PCIT for my clients. I think that PCIT can be done effectively cross culturally but perhaps there would be more buy in if there was a Māori clinician.*” (survey respondent) “*my management and the managers of partner services and organisations appear to see PCIT as a 'Pakeha intervention' best used with Pakeha*” (survey respondent) “*I was just intrigued at how negative ah the perceptions from a lot of colleagues have been about PCIT. Which is why… which is a bit hard too. And I actually just did a presentation on my, my journey of learning PCIT to the psychologists… And a lot of people came up and said *“*oh my God, how do you do that? That would be just sooo…*”* you know, they were quite kind of… interested, but just ‘that would be too hard’, and the rigorousness, and the… they felt as if that would be hard to fit into their day?*” (Group 6, clinician)
Beliefs about capabilities	• I feel more confident and competent with CDI than PDI • The detailed protocol helps me feel confident I’m doing it right	“*I never look forward to PDI1 (laughs). I always make sure I’m… really… top of my game for that and I’ll postpone it if I’m not feeling 100%.*” (Group 4, clinician) “*I haven't done PDI for a while, and it feels this big scary thing that *“*urgh I don’t want to go back to that*”*.*” (Group 4, clinician) “*I haven't done a lot of the PDI… And so therefore my confidence level has gone down. I wouldn't need a co-worker right through the process. It would be the beginning stages of PDI.*” (Group 5, clinician) “*but yeah PDI1’s always… yep (laughs). It's work, it's hard, hard work. Yep… yep… I’ve got one in two days! (laughs)*” (Group 4, clinician) “*if I had a gap now and then got back into it, it would be those initial ones that I’d really struggle with kind of confidence wise? I lean on the manual…*” (Group 3, clinician) “*I think it's definitely easier to pick this up and put into play. In, it’s that manual. There's you know, a protocol for each session… it's really easy to follow. And I think gives that clinician confidence too, you know exactly what you're doing, it's not sort of philosophical or… And even with a break, coming back to it is, is easy.*” (Group 4, clinician) “*I just like how it, you know it is manualised and it is very structured and it’s very kind of you know, if we stick to that, then you can't go wrong?*” (Group 1, clinician) “*I would feel quite happy about picking it up again, providing I had the space and place to do it. Probably because I’m a reasonably experienced clinician. I honestly would say to you, as a young clinician I don't think I would have managed some of this coaching very comfortably. I just had a lot to learn, maybe there are some clinicians that could have, but I don't think I would have had the confidence, even though it's quite manualised.*” (Group 6, clinician)
Beliefs about consequences	• I worry that time-out is harmful for children • I believe that PCIT, including the PDI phase, can make a positive difference for families	“*some of the barriers or concerns people may raise is definitely the PDI aspect. The time-out, especially with traumatized children, that kind of thing.*” (Group 5, clinician) “*[PCIT Toddler] just feels like a better fit for me. Because, like a lot of people, the time-out process is just… urgh! I know it works. I know it's good. But it kinda doesn't fit well for my personality (laughs).*” (Group 4, clinician) “*I know there's been some research around infant sleep, for example, where you know, using with young infants, leaving them to cry, that it works, but that actually if you track their physiological regulation that they actually remain very distressed but they're just not crying out? So I’m… that's what worries me personally, as I think *“*well am I just seeing…?*”” (Group 1, clinician) “*I do think [PCIT is] useful for a child when… parents really just don't have any idea about skills? They’re… like you could support them all you like, like maybe Circle of Security kind of way, but if they don't have the skills, then they just don't pick them up. (laughs) And so I think CDI is absolutely great for that…. and PDI helps a parent feel like *“*oh, I can take charge*”*, whereas previously they haven't felt like they can, and they'd either given up or otherwise they're… randomly scary which is worse…*” (Group 1, clinician) “*…it makes a difference. And that's why everyone’s so passionate, I mean who isn't passionate about PCIT? Everyone who does it, who sticks with it, loves it and is passionate about it, even despite all these things we’re saying about PDI we still all love it.*” (Group 4, clinician) “*It works. Especially CDI is like magic fairy dust, you know, making a difference for families, is… you get a real buzz from that.*” (Group 4, clinician) “*I have not experienced a therapy that can get this level of behavioural change from a child*” (survey respondent) “*…when I was able to, once I was able to, you know, that that time-out thing. Reconcile with myself that that's actually a calming, soothing kind of space to give it a go, to settle with visuals on a parent, then that's more acceptable to me? Do you know? Like, not so keen on the real kind of close the door, lock it, da da da*” (Group 6, clinician)
Memory, attention and decision processes	• There is a lot to remember, and to hold in mind. And lots of paperwork • The manual helps with this cognitive load	“*It's really paperwork heavy? Um I remember when I first started it in terms of implementing, it was really overwhelming how many bits of paper, I had to organize at the start of each session*” (Group 1, clinician) “*all of this is quite hard, right? Like you've got a lot of cognitive load anyway in these sessions… all the paperwork, and you know you’ve got to get your coding sheets, you’ve got to get all that sorted.*” (Group 1, clinician) “*you do have to focus quite hard. I found—not so much for the CDI—but you need to really be quite focused… I just… I chose a time of day and, and a time of the week, that I was quite, quite onto it.*” (Group 6, clinician) “*I remember it being quite manualised. In what you're looking for at different stages and markers to move between, between the different, different stages, as well, which is quite… I find quite helpful? It kind of gives not only us a clear plan, but like can give families markers of you know, like ‘this is what we're looking for’, ‘this is what needs to happen to move, move on’. So quite clear cut.*” (Group 4, clinician) “*a lot of it is ah, manualised, and it’s quite clear. I find you don't have to prep as much prior to a session compared to some of the other, the CBTs, the Triple Ps, those kind of interventions. So, yeah, having that sort of manualised, easy step-to-step ah process to follow.*” (Group 5, clinician)
Environmental context and resources**	• I need the right equipment for PDI to be possible, and to do it the right way • If the equipment is right, it gives me confidence—I need to be able to trust the room • Getting the right equipment is my responsibility, costs a lot, and depends on approval from managers • Virtual PCIT feels like it would be harder to control everything in PDI especially • PCIT needs to compete with other treatments in my workplace • The referrals I receive aren’t always a good fit for PCIT • I need dedicated time set aside to do PCIT	“*So having a room set up and knowing I can trust the room*” (Group 1, clinician) “*if the room, I have the room set up right, then I’m okay on my own. If I didn't have the room… I feel like that's more critical for me. If I have it all set up fine, I’m like okay. If I didn't have it set up and I had to coach in the room or something, then—urgh—yep I’m going to be less fond of this programme.*” (Group 4, clinician) “*In [agency] I think as soon as you're spending money and using up two rooms, you know there's going to, there's going to be people that ask hard questions. (laughs) And there's always going to be (pause) ideas other than PCIT as to you know where that money could go, or where those rooms could go.*” (Group 3, clinician) “*It is expensive—both in terms of the set-up, and clinician time. This is a big 'sell' for a line manager. Even though I love the work and am convinced it is valuable, it is difficult to make an argument that it is 200% more valuable (which would have to be the trade off given clinician time and initial set-up costs)*” (survey respondent) “*making sure … that people—as in the team and the manager—understand what PCIT offers and who is appropriate to be referred in. Otherwise… referrals dry out, or people just don’t think of it. Or everything goes to Circle of Security, or whatever it is… or the latest…*” (Group 4, clinician) “*Yeah. Whatever’s the latest buzz (laughs). TBRI is very popular right now, that's the buzz.*” (Group 4, clinician) “*I’ve only ever done CDI on Zoom, I’ve never done PDI. I just feel like it's too… too much could go wrong! (laughs)*” (Group 4, clinician) “*Yeah. CDI I’m comfortable with. But PDI on Zoom – oofff… So CDI – yes. But PDI on Zoom would need a bit of thinking through.*” (Group 4, clinician)
Social influences	• I feel more confident that I’m safe and doing the right thing if I have a co-worker in PDI especially • It feels more comfortable (physically and emotionally) to deliver PDI with another clinician • Additional support from supervisors is important to me • Families I work with have negative views of time-out	“*For PDI… like we would say *“*okay I’ll start with this client and do CDI, but when I get on to PDI I’m gonna need you to join me*”*. And that was, yeah I don’t know, it was just, yeah I’m not going to do PDI without another person… [Is the co-worker a practical support? … Or is it more than that?] No. No, much more than that. Yeah. Like *“*oh my God that was terr… that was hard*”*. *“*What did you think when the parent did that?*”* or *“*Did you notice the child pause then?*”* or, you know, just all that reflective stuff. Which is nice in CDI as well, but critical in PDI for me. And that probably reflects my lack of comfort with the whole PDI thing anyway. And just having another person to (laughs) go *“*oh my God that was hard*”*. (pause) But it's not like that for everybody. Some people are cool, with the PDI thing.*”* (Group 4, clinician)* “*Some of the initial PDI sessions, they are pretty full on. I mean they really are. So, you know, it's not kind of catastrophizing, it's the reality…for me to feel able to do it, and particularly as I’ve become more deskilled… somebody is kind of able to scaffold me and make sure I’m on track and if I’m finding it hard to really implement it, sort of be a bit firmer than I am. As I said, it doesn't naturally sit with me—the PDI.*” (Group 5, clinician) “*pure PCIT, including PDI, I personally struggle with it. I need somebody to work alongside me to contain me in order to be able to do it. Like, I don't like it. It doesn't really sit with my parenting philosophy*” (Group 5, clinician) “*Because if you come from Circle of Security or Tuning into Kids or Incredible Years – PCIT has a different language. And to learn the new language and to stay true to that language and not bring in the [other programmes], I think the co-working was a great method to help us integrate that.*” (Group 5, clinician) “*the minimum thing that I need personally (in PDI) to kind of process and debrief it is almost someone to just kind of reassure me that that was okay? To do. So, even though I know intellectually all the research says this works, and I know it does, and all of that. But just because of my own personal stuff, I guess. I kind of just need someone to go *“*oh that was so hard!*”*. And just to make myself feel reassured that was okay to do that, because it felt crap doing it. … that's just my personality that it's a bit of a struggle around that stuff. Unless I absolutely feel confident that there was a super good rationale for it. And I know there is, but just someone to get kind of remind me of that is handy. So even if that's just a colleague down the corridor afterwards, if I haven't had a person helping me with the actual PDI (laughs).*” (Group 4, clinician) “*Oh I think there’s emotional, you know, like you can sort of be the, the safe haven, secure base, debrief with each other, for sure… you know just making sure we're doing that for each other? So, you know, that sort of support*” (Group 6, clinician)
Emotion	• Live coaching can be stressful, but I know it is important • My co-worker helps me cope, and keeps me emotionally regulated, especially in early PDI sessions • I need to hold/contain the parent’s emotions. Families trust me that this will work and is safe, and that responsibility can be hard • I must do PCIT the right way/a certain way • I enjoy PCIT	“*But yeah, I think our own regulation needs to be considered in that process… so my reaction would have been to give up, I think, had I not had some people who were comfortable, because I didn't trust… it took me a long time to trust my ability to get [parents] through that process in a way, where I was regulated and they were regulated. And now, when I’m faced with some really difficult situations, I think *“*I’ve got this, I know how to do this*”*. And that enables me to help regulate the parent, which is what regulates the child.*” (Group 1, clinician) “*I think for those who are familiar and really on board with the full PCIT PDI, they've got a confidence, they just do it. Whereas I get, I start feeling dysregulated myself, and I’m meant to be the container for the beginning of the PDI process. So I’m kind of relying (laughs) on hands on me, in order to be able to deliver it.*” (Group 5, clinician) “*[Time-out is] an important part of the treatment—but do acknowledge that it causes me some personal discomfort. It's difficult to see/hear children distressed, even if the process is beneficial and I know it is not doing harm. Supervision issue I guess.*” (survey respondent) “*I think, knowing that we are creating a situation that is… obviously the child's… crying and screaming and you know that's, that's really hard for people to listen to on the outside.*” (Group 3, clinician) “*And there are few therapies where I think you really bring it into the room the way that you do with PCIT? And helping the person kind of sit with all of the distress that is associated with that is, seems to be really powerful and effective. I enjoy that as well.*” (Group 3, clinician) “*having someone there to also help you regulate yourself (laughs). So you’re, the parent’s regulating the child, (unclear) you're regulating the parent, and then it's sometimes quite nice—depending on who you are and your approach and your feelings about PDI—to have someone help regulate you or help regulate the child… to help keep that containment*” (Group 1, clinician) “*I’ve always… seen it as kind of building up towards that PDI1, in terms of um, I don’t know, clinician sort of worry, clinician angst about how it’s going? … it's always kind of gearing up towards that.*” (Group 5, clinician) “*I was ready for, … would be quite comfortable for the rarked up stuff. Because you know… I’m quite comfortable with the big… meltdowns. I mean I’m not saying that I enjoy them. But I, for some reason, don't get really, really triggered or distressed myself? I can keep quite calm in those situations. [Is that your experience generally …you've been a clinician for a long time, and your own parenting journey, or do you think it's a temperament thing, a personality thing—what, what do you think?] I think it's probably a bit of a mix of both.*” (Group 6, clinician) “*And, and so they'll say to me … *“*I don't want to do anything to hurt our, my relationship with my child because it's been so damaged…*”* So CDI is fine, but when we’re at the PDI they, they have those fears. And … that's a lot of pressure on us to be like *“*Trust me (laughs) I’m not going to harm your relationship with your kid*” (Group 4, clinician) “*I genuinely enjoy every session I do … compared to much of the other work I do, … it's much more immediately rewarding for me as a clinician. It also fits with my own cultural and parenting values. I love PCIT.*” (survey respondent)

Within the eight relevant domains, two were identified as being particularly salient, or influential on clinician implementation behaviour: Environmental context and resources and Social/professional role and identity. These are presented separately below; however, some data related to both domains. For example, participating clinicians spoke of the need to advocate for, and defend aspects of, PCIT with colleagues, and within the wider agency context. Advocacy was required in relation to two factors in particular. Firstly, the need for additional resources such as a second clinic room, which is typically used as an observation room or for a time-out room (Environmental context and resources). Secondly, the inclusion of time-out within the Parent-Directed Interaction phase and the acceptability of this to colleagues and managers (Social/professional role and identity).

#### Environmental Context and Resources

Clinicians regularly spoke of the importance of a suitable clinic room and equipment for PCIT, and this appeared to influence behaviour in several ways. There was a sense of there being a ‘right’ or ‘proper’ way to deliver PCIT, and a belief that this was only possible with the right equipment. Clinicians also described a sense of confidence derived from having the right room and equipment, with several noting that it was important to be able to ‘trust the room’.

“*So, having a room set up and knowing I can trust the room*” (Group 1, Clinician).

“*if the room, I have the room set up right, then I’m okay on my own. If I didn't have the room... I feel like that's more critical for me. If I have it all set up fine, I’m like, okay. If I didn't have it set up and I had to coach in the room or something, then—urgh—yep I’m going to be less fond of this programme.*” (Group 4, Clinician)

However, the cost and difficulty accessing suitable equipment, along with the extensive approvals required from agency managers was a barrier. While the barrier could be construed as relating to the setting or service, in this study it manifested as a sense of responsibility on individual clinicians for “selling” the rationale for purchases to managers, and co-ordinating the sourcing and installation of equipment, in addition to their clinical role.

“*It is expensive—both in terms of the set-up, and clinician time. This is a big 'sell' for a line manager. Even though I love the work and am convinced it is valuable, it is difficult to make an argument that it is 200% more valuable* (*which would have to be the trade-off given clinician time and initial set-up costs*)” (Survey Respondent)

Perhaps related to the COVID-19 lockdown that was in place at the time, several participants spontaneously mentioned virtual PCIT, which is an alternative that does not necessarily require the purchase of additional equipment. While virtual delivery of PCIT was not a focus of this study, it was informative that clinicians appeared to view implementation of CDI and PDI differently, even within an online context. Specifically, the Child Directed Interaction phase of PCIT was viewed as readily adaptable to an online format; however, clinicians spoke of concern that it would be difficult to conduct the PDI phase virtually, as it would be ‘harder to control’ the environment.

“*I’ve only ever done CDI on Zoom, I’ve never done PDI. I just feel like it's too… too much could go wrong! [laughs]*” (Group 4, Clinician)

“*Yeah. CDI I’m comfortable with. But PDI on Zoom—oofff! So CDI—yes. But PDI on Zoom would need a bit of thinking through*.” (Group 4, Clinician)

Participants conveyed a sense that PCIT needed to ‘compete’ with other interventions within the agency. Several clinicians were required to see children and adolescents across a wide age range, and with a variety of presenting concerns, that were not always suitable for PCIT. PCIT was seen as an intervention that requires dedicated and ‘ring-fenced’ clinician time.

“*making sure … that people—as in the team and the manager—understand what PCIT offers and who is appropriate to be referred in. Otherwise… referrals dry out, or people just don’t think of it. Or everything goes to Circle of Security, or whatever is… the latest…*” (Group 4, Clinician)

“*Yeah. Whatever’s the latest buzz [laughs]. [Trust-Based Relational Intervention] is very popular right now, that's the buzz.*” (Group 4, Clinician)

#### Social/Professional Role and Identity

There was some dissonance in clinicians’ views on time-out for children. They worried that time-out was harmful, but also believed that the PDI phase (which includes time-out) could make a profound positive difference for families (Beliefs about consequences). Clinicians were concerned about both the views of, and impact on, their colleagues who were not trained in PCIT, for example that they may hear children shouting protest during time-out and this noise may intrude on colleagues’ sessions with other clients in adjacent rooms (Beliefs about consequences). A specific concern was that colleagues may view time-out as harmful to children.

“*It absolutely would probably be the thing that I hear the most—and for myself—puts [colleagues] off. *“*I don't want to do PCIT because I hate that time-out thing*”* is what I hear. And, like other clinicians and staff freaking out with the noise of the kids screaming and banging. I mean that's a logistical issue, which is a barrier… it’s just like *“*Urgh! Do I really want to go through that [laughs], even though I know in my heart this approach works…*”” (Group 4, Clinician)

“*I mean there's a lot of… opposition to PCIT. There's a lot of *“*is this traumatic for the child*”* and just a visceral kind of like a turnoff [laughs] I think [colleagues] just don't want to be around… you know, imposing distress on a child*?” (Group 3, Clinician)

Clinicians spoke of the need to advocate for PCIT, often in isolation, to convince colleagues and managers of its safety and usefulness, given their concerns about time-out specifically. There was a strong view that having a manager who is supportive of PCIT is essential.

“*If you don't have a supportive manager it's just not going to happen… and the manager might have some really strong opinions around something like PCIT*.” (Group 3, Clinician)

“*Manager support. If you don't have that you're [stuffed].*” (Group 4, Clinician)

“*if you’re having to be the lone… you have 125% believe that this is the only way, right? [laughs] Um yeah otherwise it's like* “*okay well it's probably easier just to… do Incredible Years with this family*” *because you're having to put so much into advocating for this.*” (Group 1, Clinician)

Clinicians also spoke about a concern that PCIT was not suitable for use with indigenous Māori families. Relatedly, there was a belief that if the clinician is not Māori (i.e., Pākehā/non-Māori primarily of European descent), they should not deliver PCIT to Māori families.

“*my management and the managers of partner services and organisations appear to see PCIT as a 'Pakeha intervention' best used with Pākehā*” (Survey Respondent)

“*my culture has on occasion has been a barrier to me providing PCIT for my clients. I think that PCIT can be done effectively cross culturally but perhaps there would be more buy-in if there was a Māori clinician.*” (Survey Respondent)

The usefulness and importance of a co-worker (Social/professional role and identity; Social influences) was emphasized regularly by participants. The role of this colleague appeared to serve several functions that extended beyond providing practical support, skill development, or lessening the ‘cognitive load’ of PCIT that clinicians also referred to (Memory, attention and decision processes). Rather, it appeared to include providing tacit or implicit endorsement for delivering PCIT (Social/professional role and identity), particularly time-out within PDI.

“*the minimum thing that I need personally [in PDI] to kind of process and debrief it is almost someone to just kind of reassure me that that was okay? To do. So, even though I know intellectually all the research says this works, and I know it does, and all of that. But… I kind of just need someone to go *“*oh that was so hard!*”. *And just to make myself feel reassured that was okay to do that, because it felt [bad] doing it. … it's a bit of a struggle around that stuff. Unless I absolutely feel confident that there was a super good rationale for it. And I know there is, but just someone to get kind of remind me of that is handy*.” (Group 4, Clinician)

The co-worker also appeared to provide emotional containment/support (Emotion; Beliefs about consequences) for the clinician,

“*Oh I think there’s emotional, you know, like you can sort of be the, the safe haven, secure base, debrief with each other, for sure… you know just making sure we're doing that for each other? So, you know, that sort of support*” (Group 6, Clinician)

“*having someone there to also help you regulate yourself *(*laughs*).* So... the parent’s regulating the child, … you're regulating the parent, and then it's sometimes quite nice—depending on who you are and your approach and your feelings about PDI—to have someone help regulate you or help regulate the child… to help keep that containment*” (Group 1, Clinician)

### Identifying Behaviour Change Techniques

The Theory and Techniques online tool was used to identify behaviour change techniques associated with the relevant theoretical domains. These are presented in Table 4, along with possible (as-yet-untested) intervention components, based on this tool. The final composition of our “re-implementation” intervention will be based on several factors: the above behavioural diagnosis, an understanding of the mechanisms of action of the intervention, empirical evidence about the effects of the intervention components in other contexts, resources we have available, and practicalities and logistics. We intend to test the feasibility and acceptability of this intervention package in a clinical trial and re-iterate in future if necessary.

**Table 4:** Relevant theoretical domains, behaviour change techniques, and intervention components

Hypothesised mechanisms of action		Intervention content		
Relevant theoretical domains	Associated COM-B components (Source: Behaviour Change Wheel, Atkins et al. (2017))	Intervention functions (Source: Michie et al. (2011))	Recommended behaviour change techniques (Source: Theory and Techniques online Tool)	Possible intervention components *N.B. These are examples or illustrations of possible implementation strategies and are as-yet-untested*
Social/professional role and identity • Colleagues view time-out as harmful • Advocacy in isolation • Concern PCIT not suitable for Māori	Reflective Motivation	Training; Coercion*	*(N.B. the BCT listed in this cell have ‘inconclusive’ or marginal links between the mechanism of action and the BCT, according to the Theory and Techniques Tool)* • Social support (unspecified) • Social comparison • Credible source • Identity associated with changed behaviour	Senior Māori trainer and consultation facilitator (Credible source) Opportunity to establish cross-agency community of practice through in-person, catered meetings and weekly consultation groups (Social support; Social comparison) Encouragement to share experiences, challenges and successes with implementing PCIT (Social support; Social comparison) Remind/highlight existing position statement in support of time-out within PCIT from Aotearoa’s Ministry of Health (Credible source; Coercion*)
Environmental context and resources • Need the right equipment	Physical Opportunity	Incentivisation	• Social support (practical) • Prompts/cues • Remove aversive stimulus • Restructuring the physical environment • Restructuring the social environment • Avoidance/reducing exposure to cues for the behaviour • Adding objects to the environment	Provision of a complimentary pack of suitable toys for use in PCIT (Restructuring the physical environment; Adding objects to the environment; Prompts/cues) Provision of complimentary portable, relocatable audio-visual equipment suitable for use in PCIT (Restructuring the physical environment; Adding objects to the environment) Provision of a complimentary robust, portable time-out back-up space, that can be installed in any clinic room (Restructuring the physical environment; Adding objects to the environment)
Knowledge • Manualised protocol both helpful and restrictive • Training hard to access	Psychological Capability	Modelling; Environmental Restructuring	• Biofeedback • Instruction on how to perform behaviour • Information about antecedents • Information about health consequences • Information about social and environmental consequences	Refresher training, to include modelling of PCIT, role-plays and demonstrations (Instruction on how to perform behaviour; Information about health consequences) Provision of complimentary replacement PCIT treatment protocol and Dyadic Parent–child Interaction Coding System manual (Instruction on how to perform behaviour)
Beliefs about capabilities • Less confident with PDI	Reflective Motivation	Training; Coercion*	• Problem solving • Instruction on how to perform behaviour • Demonstration of the behaviour • Behavioural practice/rehearsal • Graded tasks • Verbal persuasion about capability • Focus on past success • Self-talk	Refresher training, to include modelling of PCIT, role-plays and demonstrations. Significant proportion of training time dedicated to the PDI phase, including problem-solving and discussion around overcoming time-out implementation challenges (Instruction on how to perform behaviour; Demonstration of the behaviour; Behavioural practice/rehearsal; Verbal persuasion about capability; Coercion)
Beliefs about consequences • PCIT, including time-out, is both helpful and harmful	Reflective Motivation	Training; Coercion*	• Information about health consequences • Salience of consequences • Information about social and environmental consequences • Anticipated regret • Information about emotional consequences • Pros and cons • Comparative imagining of future outcomes • Material incentive (behaviour) • Incentive (outcome) • Reward (outcome)	Senior PCIT clinicians to share experiences of implementation challenges and how these were overcome (Information about health consequences; Information about emotional consequences; Information about social and environmental consequences)
Memory, attention and decision processes • High cognitive load	Psychological Capability	Modelling; Environmental Restructuring	• Prompts/cues • Conserving mental resources	A shared Dropbox of resources relating to the delivery of PCIT, including client handouts, clinician prompts and summaries (Prompts/cues; Conserving mental resources)
Social influences • Co-worker is important • Supervision helpful	Social Opportunity	Education; Persuasion	• Social support (unspecified) • Social support (practical) • Social comparison • Information about others’ approval • Social reward	Complimentary access to a senior PCIT clinician who may act as an in-room co-worker for PCIT sessions, or meet to prepare for, or debrief sessions (Social support (practical); Social comparison; Information about others’ approval) Weekly PCIT consultation groups (Social support (unspecified); Social comparison)
Emotion • Stressful, but enjoyable • Support for own emotions important	Automatic Motivation	Enablement; Training	• Reduce negative emotions	PCIT co-worker (Reduce negative emotions) Weekly PCIT consultation groups (Reduce negative emotions)

^*^This term may not be self-explanatory. Michie et al., (2011) suggest that interventions incorporating coercion “could influence reflective motivation by changing conscious evaluations of the options or by establishing automatic associations between anticipation of the behaviour and negative feelings in the presence of particular cues”

## Discussion

This study used the TDF to enhance existing understanding of the influences on clinician PCIT implementation behaviour in community settings in Aotearoa post-training. The Theory and Techniques Tool was then utilised to identify several possible intervention components to support re-implementation. Clinician participants spoke frequently of the importance of a suitable clinic room, that allowed for live coaching of parents, and a time-out back-up space. Understanding the function and role of a suitable PCIT room (i.e., featuring a one-way mirror, and space for the child to be placed in time-out if required) is important. It may be that confident and/or experienced PCIT clinicians simply ‘make do’ without a room, but for inexperienced PCIT clinicians, this physical environment is particularly influential in fostering a sense of confidence. In COM-B terms, it appears to both facilitate psychological Capability, and provide physical Opportunity for implementation Behaviour to occur.

Having access to a co-worker or colleague who is familiar with PCIT, and supportive of its use, also appeared to facilitate successful implementation. The presence of this co-worker appeared to both provide social opportunity and enhance reflective motivation. Clinicians are also operating within a wider milieu where the use of time-out with children is somewhat contentious or divisive (Dadds & Tully, 2019; Lieneman & McNeil, 2023). Strong ideas of what is ‘wrong’ and ‘right’ in child behaviour management are communicated through cultural communities, social media, and professional networks. The influence of this context appears to have contributed to a wariness or discomfort about the use of time-out for some PCIT clinicians, who bring their own beliefs, values and culture as they interact with PCIT. Having access to a colleague who is supportive of PCIT appeared reassuring in this regard. Relatedly, coaching parents to use time-out was emotionally evocative for several clinicians, and their colleague appeared to provide direct or indirect scaffolding to enhance their emotion regulation. This finding is in keeping with suggestions that clinicians’ anxiety relating to delivery of exposure-based tasks (e.g., within Cognitive Behavioural Therapy-based treatments for anxiety) ought to be better accounted for in implementation research and practice (Becker-Haimes et al., 2022; Deacon & Farrell, 2013).

Clinicians also appeared to hold beliefs about what was ‘right and wrong’ in relation to the delivery of PCIT to Māori families, within Aotearoa’s unique cultural context. Non-Māori clinicians shared a belief that both engagement with families, and support for PCIT from fellow professionals would be enhanced if a Māori clinician was delivering PCIT to Māori families. This view aligns with the findings of the only study of acceptability of PCIT to Māori to date (Cargo, 2020). Also, within therapy research in general, where therapists and clients are matched on ethnicity this tends to improve both engagement and outcomes (Zane et al., 2005). If the intention is to more sustainably implement the existing PCIT protocol, then training more Māori clinicians in PCIT may be important. However, we also suggest that it is important to consider whether Māori-led development of a culturally sensitive adaptation may be more appropriate. Precedents do exist, including efforts to personalise PCIT for ethnic minorities in the USA (McCabe et al., 2020). There is considerable nuance relating to this, however. For example, a non- Māori clinician being unwilling to deliver PCIT to Māori families may inadvertently exacerbate existing disparities in care quality. Clearly it is preferable for Māori clinicians to deliver PCIT to Māori families where possible. However, training and support for non-Māori clinicians in how to deliver PCIT in a manner that upholds their responsibilities under Te Tiriti o Waitangi/the Treaty of Waitangi is important where a cultural match is not possible.

In this study, we did not elucidate the influence of clinicians’ own professional experience and training on PCIT implementation. This may be a fruitful area to explore in future research. Graduate school training and theoretical orientation (for example, comfort and familiarity with Behavioural models) may continue to exert an influence on the acceptability of programmes to clinicians many years post qualification. In Aotearoa/New Zealand, many PCIT providers are nurses, social workers, or therapists who have undertaken a 3–5 year professional training programme. It is possible that these clinicians may not have been as strongly imprinted by graduate education and/or specific models of therapy. They may be attracted to treatments that are widely accepted by their colleagues (e.g., primarily attachment-oriented approaches) and perhaps are more reliant on implicit or explicit endorsement from colleagues (hence, Social/professional role and identity) when encountering aspects of treatment (e.g., time-out) that are somewhat contentious or less acceptable.

A strength of the study is its use of well-validated theory to identify and prioritise implementation determinants, and the selection of possible intervention components, along with their hypothesized mechanisms of action (summarised in Table 4). It is distinctive in its focus on ‘re-implementation’, or supporting PCIT-trained clinicians who are rarely (or no longer) using PCIT. It ought to be noted that a clinician’s experience of training in, and implementing PCIT is highly variable, based on several factors including the agency resources they have access to. For example, some clinicians may already have access to trained co-workers but may lack suitable equipment; or lack a suitable referral stream (Timmer et al., 2016). Any re-implementation intervention needs to reflect this heterogeneity by offering a variety of possible supports. Re-implementation may also require different strategies to those associated with initial implementation. This is in keeping with literature suggesting that the interventions required to sustain delivery of a programme may differ from those required for initial adoption of the programme (Nathan et al., 2022). There is likely to be a degree of overlap, however. A recent systematic review of PCIT implementation interventions identified international studies which have incorporated remote co-therapy (Funderburk et al., 2015) and learning collaboratives (Herschell et al., 2015) to support the initial post-training adoption of PCIT by clinicians (Woodfield et al., 2022).

There were several limitations to the study. Utilising focus groups as a methodology may have introduced a social desirability bias, or tendency for participants to agree with each other, possibly inflating the salience of a particular domain. Also, focus groups were facilitated by a PCIT within-programme trainer (MW) who was known to most participants, and this may have influenced or possibly limited what participants chose to share. Also, while this is perhaps justifiable given our interest in clinician experiences, the perspectives of health care consumers (families and/or children) were not included. Their attitudes toward, for example, time-out or being seen by a clinician from a different culture, would have enhanced our understanding (cf. clinicians sharing their *perceptions* of acceptability to families). This was not a co-design study; however, the introduction of a member checking process, or presenting the proposed intervention components to participating clinicians would have likely enhanced our understanding of the acceptability and feasibility of these—although we do plan to pilot the intervention to determine this more systematically. Also, it would have been useful to gather data relating to the clinicians’ own experiences of being parented, whether they are parents themselves, and their parenting values. If a clinician has limited clinical experience, they are perhaps more likely to base their Beliefs about Consequences, for example, on their personal experience (e.g., “I didn’t use time-out with my children, so time-out is not necessary in PCIT”).

To our knowledge, this is the first study to use the TDF to explore influences on the sustained implementation of PCIT and is the first to propose associated intervention components. Possible components of a re-implementation intervention include refresher training with a senior Māori trainer, access to a mobile PCIT co-worker, provision of suitable audio-visual equipment, provision of a portable time-out space, regular group consultation, and access to materials to support PCIT delivery including material to facilitate the use of ‘swoop and go’ (McNeil & Hembree-Kigin, 2011) as an alternative to the use of a time-out room. Our intention is to pilot this intervention package and determine the feasibility and acceptability to clinicians, and we have described the parameters of this randomised, controlled trial in detail elsewhere (Woodfield et al., 2023).

With the concept of re-implementation, we are seeking to draw attention to the rich resource of already-trained clinicians to whom PCIT is acceptable, and who have a desire to deliver PCIT despite changed circumstances or inadequate supports. Stated plainly, re-implementation might represent what pre-implementation should have been, but was not. There has been considerable investment into the training of PCIT clinicians in Aotearoa/New Zealand, and it makes good sense to support already-trained, willing clinicians to resume delivery of this effective treatment to children and families in need.

### Supplementary Information

Below is the link to the electronic supplementary material.

Supplementary file1 (DOC 62 KB)

## Declarations

### Methods and Reporting Guidelines

We followed Atkins and colleagues’ 2017 paper (“A guide to using the Theoretical Domains Framework of behaviour change to investigate implementation problems”) in the design and execution of the study. We have described the study according to Tong and colleagues’ 2007 reporting guidelines, namely the Consolidated Criteria for Reporting Qualitative Research (COREQ).

## References

[CR1] Advisory Group on Conduct Problems (2011). Conduct problems: Effective programmes for 8–12 year olds.

[CR2] Atkins L, Francis J, Islam R, O’Connor D, Patey A, Ivers N, Foy R, Duncan EM, Colquhoun H, Grimshaw JM, Lawton R, Michie S (2017). A guide to using the Theoretical Domains Framework of behaviour change to investigate implementation problems. Implementation Science.

[CR3] Barker F, Atkins L, de Lusignan S (2016). Applying the COM-B behaviour model and behaviour change wheel to develop an intervention to improve hearing-aid use in adult auditory rehabilitation. International Journal of Audiology.

[CR4] Bauer MS, Kirchner J (2020). Implementation science: What is it and why should I care?. Psychiatry Research.

[CR5] Becker-Haimes EM, Klein CC, Frank HE, Oquendo MA, Jager-Hyman S, Brown GK, Brady M, Barnett ML (2022). Clinician maladaptive anxious avoidance in the context of implementation of evidence-based interventions: A commentary [hypothesis and theory]. Frontiers in Health Services.

[CR6] Brierley ML, Smith LR, Bailey DP, Every SA, Staines TA, Chater AM (2021). Perceived influences on reducing prolonged sitting in police staff: A qualitative investigation using the Theoretical Domains Framework and COM-B model. BMC Public Health.

[CR7] Cane J, O’Connor D, Michie S (2012). Validation of the theoretical domains framework for use in behaviour change and implementation research. Implementation Science.

[CR8] Carey RN, Connell LE, Johnston M, Rothman AJ, De Bruin M, Kelly MP, Michie S (2019). Behavior change techniques and their mechanisms of action: a synthesis of links described in published intervention literature. Annals of Behavioral Medicine.

[CR9] Cargo T (2020). Kei te whetewhete mai ki ngā matua—Parent Whispering. PCIT: The art of empowering parents to change to improve tamariki behaviour.

[CR10] Castro O, Vergeer I, Bennie J, Cagas J, Biddle SJ (2021). Using the behavior change wheel to understand university students’ prolonged sitting time and identify potential intervention strategies. International Journal of Behavioral Medicine.

[CR11] Clarke V, Braun V (2013). Successful qualitative research: A practical guide for beginners.

[CR12] Coghill D (2013). Editorial: Do clinical services need to take conduct disorder more seriously?. Journal of Child Psychology and Psychiatry and Allied Disciplines.

[CR13] Connell LE, Carey RN, De Bruin M, Rothman AJ, Johnston M, Kelly MP, Michie S (2019). Links between behavior change techniques and mechanisms of action: An expert consensus study. Annals of Behavioral Medicine.

[CR14] Courtenay M, Rowbotham S, Lim R, Peters S, Yates K, Chater A (2019). Examining influences on antibiotic prescribing by nurse and pharmacist prescribers: a qualitative study using the Theoretical Domains Framework and COM-B. British Medical Journal Open.

[CR15] Dadds M, Tully LA (2019). What is it to discipline a child: What should it be? A reanalysis of time-out from the perspective of child mental health, attachment, and trauma. American Psychologist.

[CR16] Damschroder LJ, Reardon CM, Widerquist MAO, Lowery J (2022). The updated Consolidated Framework for Implementation Research based on user feedback. Implementation Science.

[CR17] Deacon BJ, Farrell NR, Storch EA, McKay D (2013). Therapist barriers to the dissemination of exposure therapy. Handbook of treating variants and complications in anxiety disorders.

[CR18] Eyberg SM (1988). Parent-child interaction therapy: Integration of traditional and behavioral concerns. Child and Family Behavior Therapy.

[CR19] Eyberg SM, Funderburk B (2011). Parent–child interaction therapy protocol.

[CR20] Funderburk B, Chaffin M, Bard E, Shanley J, Bard D, Berliner L (2015). Comparing client outcomes for two evidence-based treatment consultation strategies. Journal of Clinical Child and Adolescent Psychology.

[CR21] Gillies K, Brehaut J, Coffey T, Duncan EM, Francis JJ, Hey SP, Presseau J, Weijer C, Campbell MK (2021). How can behavioural science help us design better trials?. Trials.

[CR22] Gould GS, Bar-Zeev Y, Bovill M, Atkins L, Gruppetta M, Clarke MJ, Bonevski B (2017). Designing an implementation intervention with the Behaviour Change Wheel for health provider smoking cessation care for Australian Indigenous pregnant women. Implementation Science.

[CR23] Herlitz L, MacIntyre H, Osborn T, Bonell C (2020). The sustainability of public health interventions in schools: A systematic review. Implementation Science.

[CR24] Herschell AD, Kolko DJ, Scudder AT, Taber-Thomas S, Schaffner KF, Hiegel SA, Iyengar S, Chaffin M, Mrozowski S (2015). Protocol for a statewide randomized controlled trial to compare three training models for implementing an evidence-based treatment. Implementation Science.

[CR25] Herschell AD, Schaffner KF, Taber-Thomas S, Scudder AT, Niec LN (2018). Getting parent–child interaction therapy to scale. Handbook of parent–child interaction therapy: Innovations and applications for research and practice.

[CR26] Johnston M, Carey RN, Connell Bohlen LE, Johnston DW, Rothman AJ, de Bruin M, Kelly MP, Groarke H, Michie S (2021). Development of an online tool for linking behavior change techniques and mechanisms of action based on triangulation of findings from literature synthesis and expert consensus. Translational Behavioral Medicine.

[CR27] Kaplan, H. C., & Walsh, K. E. (2022). Context in implementation science. *Pediatrics, 149*(Suppl 3). 10.1542/peds.2020-045948C 10.1542/peds.2020-045948C 35230429

[CR28] Kierkegaard P, Hicks T, Allen AJ, Yang Y, Hayward G, Glogowska M, Nicholson BD, Buckle P (2021). Strategies to implement SARS-CoV-2 point-of-care testing into primary care settings: A qualitative secondary analysis guided by the Behaviour Change Wheel. Implementation Science Communications.

[CR29] Lewis CC, Boyd MR, Walsh-Bailey C, Lyon AR, Beidas R, Mittman B, Aarons GA, Weiner BJ, Chambers DA (2020). A systematic review of empirical studies examining mechanisms of implementation in health. Implementation Science.

[CR30] Lewis CC, Klasnja P, Powell BJ, Lyon AR, Tuzzio L, Jones S, Walsh-Bailey C, Weiner B (2018). From classification to causality: Advancing understanding of mechanisms of change in implementation science. Frontiers in Public Health.

[CR31] Lieneman C, McNeil CB (2023). Time-out for child behavior management.

[CR32] Lieneman CC, Brabson LA, Highlander A, Wallace NM, McNeil CB (2017). Parent–child interaction therapy: Current perspectives. Psychology Research and Behavior Management.

[CR33] Lorencatto F, Rapport F, Clay-Williams R, Braithwaite J (2022). The theoretical domains framework. Implementation science: The key concepts.

[CR34] Manatū Hauora/Ministry of Health. (2020). *Te Tiriti o Waitangi Framework*.

[CR35] McCabe, K. M., Yeh, M., & Zerr, A. A. (2020). Personalizing behavioral parent training interventions to improve treatment engagement and outcomes for culturally diverse families. *Psychology Research and Behavior Management*, 41–53. 10.2147/PRBM.S230005 10.2147/PRBM.S230005 PMC6966146 32021508

[CR36] McNeil CB, Hembree-Kigin TL (2011). Parent–child interaction therapy.

[CR37] Michie S, Atkins L, West R (2014). The behaviour change wheel: A guide to designing interventions.

[CR38] Michie S, van Stralen MM, West R (2011). The behaviour change wheel: A new method for characterising and designing behaviour change interventions. Implementation Science.

[CR39] Mutu M (2019). The treaty claims settlement process in New Zealand and its impact on Māori. Land.

[CR40] Nathan N, Powell BJ, Shelton RC, Laur CV, Wolfenden L, Hailemariam M, Yoong SL, Sutherland R, Kingsland M, Waltz TJ, Hall A (2022). Do the Expert Recommendations for Implementing Change (ERIC) strategies adequately address sustainment?. Frontiers in Health Services.

[CR41] O’Connor C, Joffe H (2020). Intercoder reliability in qualitative research: Debates and practical guidelines. International Journal of Qualitative Methods.

[CR42] Patey AM, Islam R, Francis JJ, Bryson GL, Grimshaw JM (2012). Anesthesiologists’ and surgeons’ perceptions about routine pre-operative testing in low-risk patients: application of the Theoretical Domains Framework (TDF) to identify factors that influence physicians’ decisions to order pre-operative tests. Implementation Science.

[CR43] PCIT International Inc (2018). Training requirements for certification as a PCIT therapist.

[CR44] Presseau J, McCleary N, Lorencatto F, Patey AM, Grimshaw JM, Francis JJ (2019). Action, actor, context, target, time (AACTT): A framework for specifying behaviour. Implementation Science.

[CR45] Sales AE, Barnaby DP, Rentes VC (2021). Letter to the editor on “the implementation research logic model: a method for planning, executing, reporting, and synthesizing implementation projects” (Smith JD, Li DH, Rafferty MR. the implementation research logic model: a method for planning, executing, reporting, and synthesizing implementation projects. Implement Sci. 2020;15 (1):84. Doi:10.1186/s13012-020-01041-8). Implementation Science.

[CR46] Schleider JL, Beidas RS (2022). Harnessing the Single-Session Intervention approach to promote scalable implementation of evidence-based practices in healthcare. Frontiers in Health Services.

[CR47] Scott S, Gardner F, Thapar A, Pine DS, Leckman JF, Scott S, Snowling MJ, Taylor E (2015). Parenting programs. Rutter's child and adolescent psychiatry.

[CR48] Shoesmith A, Hall A, Wolfenden L, Shelton RC, Powell BJ, Brown H, McCrabb S, Sutherland R, Yoong S, Lane C (2021). Barriers and facilitators influencing the sustainment of health behaviour interventions in schools and childcare services: A systematic review. Implementation Science.

[CR49] Statistics New Zealand. (2021). *Māori population estimates: At 30 June 2021*. https://www.stats.govt.nz/information-releases/maori-population-estimates-at-30-june-2021

[CR50] Thomas, R., Abell, B., Webb, H. J., Avdagic, E., & Zimmer-Gembeck, M. J. (2017). Parent–child interaction therapy: A meta-analysis. *Pediatrics, 140*(3). 10.1542/peds.2017-0352 10.1542/peds.2017-0352 28860132

[CR51] Timmer SG, Urquiza AJ, Boys DK, Forte LA, Quick-Abdullah D, Chan S, Gould W (2016). Filling potholes on the implementation highway: Evaluating the implementation of Parent-Child Interaction Therapy in Los Angeles County. Child Abuse Neglect.

[CR52] Vasileva M, Graf RK, Reinelt T, Petermann U, Petermann F (2020). Research review: A meta-analysis of the international prevalence and comorbidity of mental disorders in children between 1 and 7 years. Journal of Child Psychology and Psychiatry.

[CR53] Ward MA, Theule J, Cheung K (2016). Parent-child interaction therapy for child disruptive behaviour disorders: A meta-analysis. Child and Youth Care Forum.

[CR54] Weiner BJ, Lewis CC, Sherr K (2022). Introducing implementation science.

[CR55] West R, Michie S (2020). A brief introduction to the COM-B Model of behaviour and the PRIME Theory of motivation [v1]. Qeios.

[CR56] Woodfield MJ, Cargo T, Barnett D, Lambie I (2020). Understanding New Zealand therapist experiences of Parent–Child Interaction Therapy (PCIT) training and implementation, and how these compare internationally. Children and Youth Services Review.

[CR57] Woodfield MJ, Cargo T, Merry S, Hetrick SE (2023). Protocol for a randomised pilot study of a novel Parent–Child Interaction Therapy (PCIT) ‘re-implementation’ intervention. Pilot and Feasibility Studies.

[CR58] Woodfield, M. J., Cargo, T., Merry, S. N., & Hetrick, S. E. (2021). Barriers to Clinician Implementation of Parent–Child Interaction Therapy (PCIT) in New Zealand and Australia: What Role for Time-Out? *International Journal of Environmental Research and Public Health, 18*(24), 13116. https://www.mdpi.com/1660-4601/18/24/13116 10.3390/ijerph182413116 PMC8700887 34948725

[CR59] Woodfield MJ, Merry S, Hetrick SE (2022). Clinician adoption of Parent–Child Interaction Therapy: A systematic review of implementation interventions. Implementation Research and Practice.

[CR60] Zane N, Sue S, Chang J, Huang L, Huang J, Lowe S, Srinivasan S, Chun K, Kurasaki K, Lee E (2005). Beyond ethnic match: Effects of client-therapist cognitive match in problem perception, coping orientation, and therapy goals on treatment outcomes. Journal of Community Psychology.

[CR61] Zimmer-Gembeck MJ, Kerin JL, Webb HJ, Gardner AA, Campbell SM, Swan K, Timmer SG (2018). Improved perceptions of emotion regulation and reflective functioning in parents: Two additional positive outcomes of parent-child interaction therapy. Behavior Therapy.

